# Open and closed conformations of two SpoIIAA-like proteins (YP_749275.1 and YP_001095227.1) provide insights into membrane association and ligand binding

**DOI:** 10.1107/S1744309109042481

**Published:** 2009-12-08

**Authors:** Abhinav Kumar, Andrei Lomize, Kevin K. Jin, Dennis Carlton, Mitchell D. Miller, Lukasz Jaroszewski, Polat Abdubek, Tamara Astakhova, Herbert L. Axelrod, Hsiu-Ju Chiu, Thomas Clayton, Debanu Das, Marc C. Deller, Lian Duan, Julie Feuerhelm, Joanna C. Grant, Anna Grzechnik, Gye Won Han, Heath E. Klock, Mark W. Knuth, Piotr Kozbial, S. Sri Krishna, David Marciano, Daniel McMullan, Andrew T. Morse, Edward Nigoghossian, Linda Okach, Ron Reyes, Christopher L. Rife, Natasha Sefcovic, Henry J. Tien, Christine B. Trame, Henry van den Bedem, Dana Weekes, Qingping Xu, Keith O. Hodgson, John Wooley, Marc-André Elsliger, Ashley M. Deacon, Adam Godzik, Scott A. Lesley, Ian A. Wilson

**Affiliations:** a Joint Center for Structural Genomics, http://www.jcsg.org, USA; b Stanford Synchrotron Radiation Lightsource, SLAC National Accelerator Laboratory, Menlo Park, CA, USA; c Department of Medicinal Chemistry, College of Pharmacy, University of Michigan, Ann Arbor, MI, USA; d Department of Molecular Biology, The Scripps Research Institute, La Jolla, CA, USA; e Center for Research in Biological Systems, University of California, San Diego, La Jolla, CA, USA; f Program on Bioinformatics and Systems Biology, Burnham Institute for Medical Research, La Jolla, CA, USA; g Protein Sciences Department, Genomics Institute of the Novartis Research Foundation, San Diego, CA, USA; h Photon Science, SLAC National Accelerator Laboratory, Menlo Park, CA, USA

**Keywords:** YP_001095227.1, YP_749275.1, SpoIIAA-like proteins

## Abstract

The crystal structures of the proteins encoded by the YP_749275.1 and YP_001095227.1 genes from *Shewanella frigidimarina* and *S. loihica*, respectively, have been determined at 1.8 and 2.25 Å resolution, respectively. These proteins are members of a novel family of bacterial proteins that adopt the α/β SpoIIAA-like fold found in STAS and CRAL-TRIO domains. Despite sharing 54% sequence identity, these two proteins adopt distinct conformations arising from different dispositions of their α2 and α3 helices. In the ‘open’ conformation (YP_001095227.1), these helices are 15 Å apart, leading to the creation of a deep nonpolar cavity. In the ‘closed’ structure (YP_749275.1), the helices partially unfold and rearrange, occluding the cavity and decreasing the solvent-exposed hydrophobic surface. These two complementary structures are reminiscent of the conformational switch in CRAL-TRIO carriers of hydrophobic compounds. It is suggested that both proteins may associate with the lipid bilayer in their ‘open’ monomeric state by inserting their amphiphilic helices, α2 and α3, into the lipid bilayer. These bacterial proteins may function as carriers of nonpolar substances or as interfacially activated enzymes.

## Abstract 2

The crystal structures of two orthologous proteins from different *Shewanella* species have uncovered a resemblance to CRAL-TRIO carrier proteins, which suggest that they function as transporters of small nonpolar molecules. One protein adopts an open conformation, while the other adopts a closed structure that may act as a conformational switch in the transport of ligands at the membrane surface.

## 1. Introduction

The YP_749275.1 gene from *Shewanella frigidimarina* encodes a protein of unknown function with a molecular weight of 14 502 Da (residues 1–126) and a calculated isoelectric point of 4.9. An ortholog with 54% sequence identity from *S. loihica* (YP_001095227.1) is also of unknown function, with a molecular weight of 14 105 Da (residues 1–125) and a calculated isoelectric point of 4.9.

Both sequences have been assigned to a family of 119 bacterial and archaeal proteins (PB000640) in the automatically generated Pfam-B entries (Finn *et al.*, 2008 ▶). The proteins in the PB000640 family are composed of a single domain, with one exception which is fused to a universal stress protein (UspA) domain. Profile–profile sequence-comparison methods (Jaroszewski *et al.*, 2005 ▶) detected distant homology to proteins which adopt the SpoIIAA-like fold (Kovacs *et al.*, 1998 ▶). These NTP-binding proteins are involved in regulating the sporulation sigma factor F in *Bacillus subtilis*. However, since this relationship is relatively distant (<15% sequence identity), it does not allow a direct functional inference.

Here, we report the structures of these two orthologs determined using the semi-automated high-throughput pipeline of the Joint Center for Structural Genomics (JCSG; Lesley *et al.*, 2002 ▶) as part of the Protein Structure Initiative of the National Institute of General Medical Sciences (http://www.nigms.nih.gov/Initiatives/PSI/).

Both proteins have now been classified by *SCOP* (Hubbard *et al.*, 1999 ▶) as being members of a novel Sfri0576-like family which belongs to the SpoIIAA superfamily. However, despite sharing the same fold, their structures differ significantly in the relative disposition of two surface α-helices and in their mode of dimerization. The arrangement of the α-helices suggests that the proteins may associate with the membrane and possibly function as carriers of nonpolar compounds similar to CRAL-TRIO domains.

## 2. Materials and methods

### 2.1. Protein production and crystallization

The clones for YP_749275.1 and YP_001095227.1 were generated using the Polymerase Incomplete Primer Extension (PIPE) cloning method (Klock *et al.*, 2008 ▶). The gene encoding YP_749275.1 (Gen­Bank YP_749275; gi:114561762; Swiss-Prot Q087X8) was amplified from *S. frigidimarina* NCIMB 400 genomic DNA using *PfuTurbo* DNA polymerase (Stratagene) and I-PIPE (Insert) primers (forward primer, 5′-ctgtacttccagggcATGGATATGAAGAAACATGGTTTA­TCG-3′; reverse primer, 5′-aattaagtcgcgttaATATCGAAGCCATTT­CAAGG­CGTCATC-3′; target sequence in upper case) that included sequences for the predicted 5′ and 3′ ends. The expression vector pSpeedET, which encodes an amino-terminal tobacco etch virus (TEV) protease-cleavable expression and purification tag (MGSD­KIHHHHHHENLYFQ/G), was PCR-amplified with V-PIPE (Vector) primers (forward primer, 5′-taacgcgacttaattaactcgtttaaacggtctccagc-­3′; reverse primer, 5′-gccctggaagtacaggttttcgtgatgatgatgatg­atg-3′). V-­PIPE and I-PIPE PCR products were mixed to anneal the amplified DNA fragments together. *Escherichia coli* GeneHogs (Invitro­gen) com­petent cells were transformed with the V-PIPE/I-­PIPE mixture and dispensed onto selective LB–agar plates. The cloning junctions were confirmed by DNA sequencing. Expression was performed in selenomethionine-containing medium at 310 K with suppression of normal methionine synthesis (Van Duyne *et al.*, 1993 ▶). At the end of fermentation, lysozyme was added to the culture to a final concentration of 250 µg ml^−1^ and the cells were harvested and frozen. After one freeze–thaw cycle, the cells were homogenized in lysis buffer [50 m*M* HEPES pH 8.0, 50 m*M* NaCl, 10 m*M* imidazole, 1 m*M* tris(2-­carboxyethyl)phosphine–HCl (TCEP)] and passed through a Microfluidizer (Microfluidics). The lysate was clarified by centrifugation at 32 500*g* for 30 min and loaded onto nickel-chelating resin (GE Healthcare) pre-equilibrated with lysis buffer; the resin was washed with wash buffer [50 m*M* HEPES pH 8.0, 300 m*M* NaCl, 40 m*M* imidazole, 10%(*v*/*v*) glycerol, 1 m*M* TCEP] and the protein was eluted with elution buffer [20 m*M* HEPES pH 8.0, 300 m*M* imidazole, 10%(*v*/*v*) glycerol, 1 m*M* TCEP]. The eluate was buffer-exchanged with TEV buffer (20 m*M* HEPES pH 8.0, 200 m*M* NaCl, 40 m*M* imidazole, 1 m*M* TCEP) using a PD-10 column (GE Healthcare) and incubated with 1 mg TEV protease per 15 mg of eluted protein. The protease-treated eluate was run over nickel-chelating resin (GE Healthcare) pre-equilibrated with HEPES crystallization buffer (20 m*M* HEPES pH 8.0, 200 m*M* NaCl, 40 m*M* imidazole, 1 m*M* TCEP) and the resin was washed with the same buffer. The flowthrough and wash fractions were combined and concentrated to 17.7 mg ml^−1^ by centrifugal ultrafiltration (Millipore) for crystallization trials and crystallized by mixing 200 nl protein with 200 nl crystallization solution in sitting drops above a 50 µl reservoir volume using the nanodroplet vapor-diffusion method (Santarsiero *et al.*, 2002 ▶) with standard JCSG crystallization protocols (Lesley *et al.*, 2002 ▶). The crystallization reagent for YP_749275.1 consisted of 0.2 *M* calcium acetate and 20.0% PEG 3350. A diamond-shaped crystal of approximate dimensions 50 × 50 × 50 µm was harvested after 10 d at 277 K. Ethylene glycol was added to the crystal as a cryoprotectant to a final concentration of 8%(*v*/*v*). Initial screening for diffraction was carried out using the Stanford Automated Mounting system (SAM; Cohen *et al.*, 2002 ▶) at the Stanford Synchrotron Radiation Lightsource (SSRL, Menlo Park, California, USA). The diffraction data were indexed in monoclinic space group *C*2. The oligomeric state of YP_749275.1 in solution was determined using a 1 × 30 cm Superdex 200 column (GE Healthcare) coupled with miniDAWN static light-scattering and Optilab differential refractive-index detectors (SEC/SLS; Wyatt Technology). The mobile phase consisted of 20 m*M* Tris pH 8.0, 150 m*M* sodium chloride and 0.02%(*w*/*v*) sodium azide. The molecular weight was calculated using the *ASTRA* v.5.1.5 software (Wyatt Technology).

The gene encoding YP_001095227.1 (GenBank YP_001095227; gi:127514030; Swiss-Prot A3QHM0) was amplified by polymerase chain reaction (PCR) from *Shewanella* sp. PV-4 genomic DNA using *PfuTurbo* DNA polymerase (Stratagene) and I-PIPE (Insert) primers (forward primer, 5′-ctgtacttccagggcATGAGCAGCAATCTACATG­GTATCGCC-3′; reverse primer, 5′-aattaagtcgcgttaACACAGCCA­GTCGAGTGCATCGCGT-3′; target sequence in upper case) that included sequences for the predicted 5′ and 3′ ends. Cloning, expression and purification were performed as described for YP_749275.1. Purified YP_001095227.1 was concentrated to 17.4 mg ml^−1^ by centrifugal ultrafiltration (Millipore) for crystallization trials. YP_001095227.1 was crystallized using the nanodroplet vapor-diffusion method as described for YP_749275.1. The crystallization reagent for YP_001095227.1 consisted of 0.2 *M* NaCl, 37% 2-methyl-2,4-pentanediol and 0.1 *M* Tris pH 7.33. A rod-shaped crystal of approximate dimensions 70 × 30 × 300 µm was harvested after 36 days at 277 K. Initial screening for diffraction was carried out as described for YP_749275.1. The diffraction data were indexed in ortho­rhombic space group *C*222_1_. The oligomeric state of YP_001095227.1 in solution was determined using a 0.8 × 30 cm Shodex Protein KW-­803 column (Thomson Instruments) pre-calibrated with gel-filtration standards (Bio-Rad).

### 2.2. Data collection, structure solution and refinement

For YP_749275.1 and YP_001095227.1, selenium multiwavelength anomalous diffraction (MAD) data were collected on beamline 11-1 at SSRL at wavelengths corresponding to the inflection, high-energy remote and peak. The data sets were collected at 100 K using a MAR 325 CCD detector and the *BLU-ICE* data-collection environment (McPhillips *et al.*, 2002 ▶). The MAD data were integrated using *MOSFLM* (Leslie, 1992 ▶) and scaled with the program *SCALA* from the *CCP*4 suite (Collaborative Computational Project, Number 4, 1994 ▶). Phasing was performed with *SHELXD* (Schneider & Sheldrick, 2002 ▶) and *autoSHARP* (Bricogne *et al.*, 2003 ▶), which resulted in a mean figure of merit of 0.35 to 1.8 Å resolution for YP_749275.1 with five selenium sites and of 0.43 to 2.25 Å resolution for YP_001095227.1 with six selenium sites. Automatic model building was performed with *ARP*/*wARP* (Cohen *et al.*, 2004 ▶). Model completion and refinement were performed with *Coot* (Emsley & Cowtan, 2004 ▶) and *REFMAC*5.2 (Winn *et al.*, 2003 ▶) using the inflection-wavelength data. The refinement of YP_749275.1 included experimental phase restraints in the form of Hendrickson–Lattman coefficients from *SHARP* and TLS refinement with one TLS group per chain. The refinement of YP_001095227.1 included experimental phase restraints, NCS restraints (positional weight 0.5 and thermal weight 2.0) and TLS refinement with one TLS group per chain. Data-collection and refinement statistics are summarized in Table 1 ▶.

### 2.3. Validation and deposition

**Figure 1:**
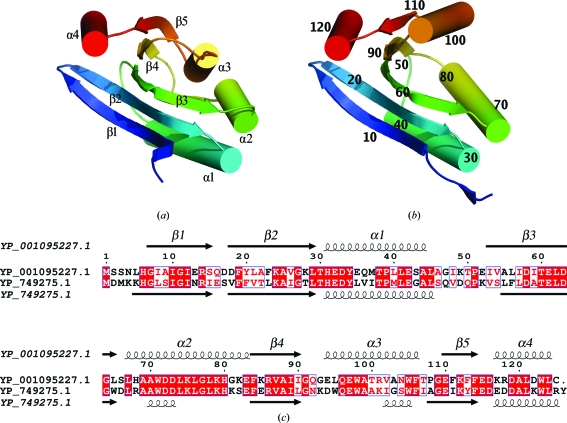
Crystal structures of (*a*) YP_749275.1 (PDB code 2ook) and (*b*) YP_001095227.1 (PDB code 2q3l) shown as ribbon diagrams of protein monomers color-coded from the N-­terminus (blue) to the C-terminus (red). Helices α1–α4 and strands β1–β5 are indicated for YP_749275.1, while every tenth residue is numbered for YP_001095227.1. (*c*) Diagram showing the secondary-structure elements of YP_749275.1 and YP_001095227.1 superimposed on their primary sequences. Strands and helices are indicated by arrows and coils, respectively, and labeled sequentially as β1, β2 *etc*. and α1, α2 *etc*. Identical residues in these proteins are shown in white on a red background, while similar residues are shown as the reverse (red on a white background).

The quality of the crystal structure was analyzed using the JCSG quality-control server. This server verifies the stereochemical quality of the model using *AutoDepInputTool* (Yang *et al.*, 2004 ▶), *MolProbity* (Davis *et al.*, 2007 ▶), *WHATIF* (Vriend, 1990 ▶) and *RESOLVE* (Terwilliger, 2003 ▶), as well as several in-house scripts, and summarizes the outputs. Protein quaternary-structure analysis was carried out using the *PISA* server (Krissinel & Henrick, 2007 ▶). Fig. 1 ▶(*c*) was adapted from *ESPript* (Gouet *et al.*, 1999 ▶) and all other figures were prepared with *PyMOL* (DeLano Scientific). The atomic coordinates and experimental structure factors for YP_749275.1 and YP_001095227.1 have been deposited in the PDB under codes 2ook and 2q3l, respectively.

### 2.4. Orientation of proteins in membranes

The spatial orientations of membrane-associated proteins with respect to the lipid bilayer, including the maximal penetration depths (*D*) of protein residues in the hydrocarbon core and the free energy of transfer of the protein from water to the membrane (Δ*G*_transf_), were calculated using the *PPM* program, as previously described by Lomize *et al.* (2006 ▶). Two major contributions are considered in the current version of *PPM*: (i) the water–lipid transfer energy calculated with atomic solvation parameters (favorable for nonpolar C and S atoms and unfavorable for polar N and O atoms) and (ii) the de­ionization penalty for charged residues. The smoothing function with a decay parameter of 1 Å was used to describe the gradual polarity changes at the lipid head group–hydrocarbon core boundary.

## 3. Results

### 3.1. Overall structure

The crystal structures of YP_749275.1 and YP_001095227.1 (Fig. 1 ▶) were determined independently by the MAD method to 1.80 and 2.25 Å resolution, respectively. Data-collection and refinement statistics are summarized in Table 1 ▶. The final model of YP_749275.1 consists of a protein dimer (residues 2–126 for chain *A* and residues 3–79 and 82–126 for chain *B*), six ethylene glycols and 224 water molecules in the asymmetric unit. Similarly, the structure of YP_001095227.1 consists of a protein dimer (residues 0–125 for chain *A* and residues 0–48 and 53–125 for chain *B*), two sodium ions, two chloride ions, nine 2-methyl-2,4-pentanediol molecules and 95 water molecules in the asymmetric unit.

The Matthews coefficient (*V*_M_; Matthews, 1968 ▶) and estimated solvent content are 2.16 Å^3^ Da^−1^ and 43.0% for YP_749275.1, and 2.63 Å^3^ Da^−1^ and 53.3% for YP_001095227.1. The Ramachandran plot produced by *MolProbity* shows that 98.8% (YP_749275.1) and 97.5% (YP_001095227.1) of the residues are in favored regions, with no outliers.

YP_749275.1 and YP_001095227.1 both adopt the SpoIIAA-like fold according to *SCOP* and *CATH* (Cuff *et al.*, 2009 ▶). This fold consists of four turns of β/α superhelix with an additional N-terminal β-strand. The five strands and four helices are arranged in the order β1–β2–α1–β3–α2–β4–α3–β5–α4. The two proteins share the same topology (Fig. 1 ▶), although some noticeable differences are apparent in the lengths of the β-strands and α-helices.

### 3.2. Comparison of the YP_749275.1 and YP_001095227.1 structures

**Figure 2:**
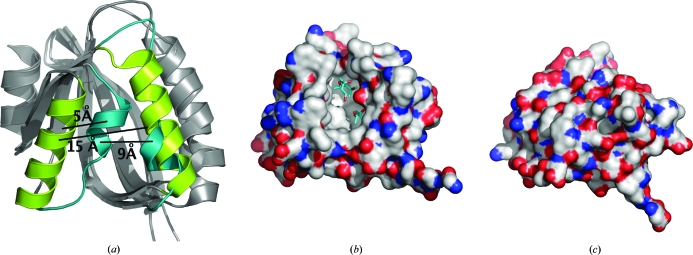
Structural comparison between YP_749275.1 and YP_001095227.1. (*a*) Superposition of the two structures underscores their overall similarity. The major differences arise from the different relative positions and orientations of helices α2 and α3. These two helices in YP_749275.1 (cyan) are close together, while they separate in YP_001095227.1 (green). (*b*) A surface representation of YP_001095227.1 in the ‘open’ state shows the presence of a wide cavity. The C atoms are colored grey, O atoms red and N atoms blue. The 2-methyl-2,4-pentanediol (MPD) molecules are shown as cyan and red sticks. The opening and closing of the cavity is regulated by the movement of helix α3 away from or towards α2, which involves an ∼5 Å translation and a 90° rotation of this helix. (*c*) YP_749275.1 reveals no cavity in the ‘closed’ state of the protein.

Despite sharing high sequence identity (54%), the two structures align with an overall r.m.s.d. of 4.2 Å (123 aligned C^α^ atoms). However, most of the deviations occur around the α2 and α3 helices (Fig. 2 ▶ *a*). The r.m.s.d. decreases to 1.6 Å over 92 C^α^ atoms when these two helices are excluded from the alignment.

YP_001095227.1 displays an ‘open’ conformation in which the α2 and α3 helices are 15 Å apart and form either side of a large channel that runs across one face of the protein (Figs. 2 ▶ *a* and 2 ▶ *b*). An analysis using the *CastP* server (Binkowski *et al.*, 2003 ▶) reveals a deep cavity (1743 Å^3^) lined by over 20 residues that are all hydrophobic, except for Asp73, which is hydrogen bonded to Tyr34. The floor of the cavity is formed by the β-sheet and helix α1. This large hydrophobic cavity represents a potential ligand-binding pocket that is occupied in the crystal structure by three 2-methyl-2,4-pentanediol (MPD) molecules, which are likely to stabilize the ‘open’ conformation by partially filling the cavity.

In contrast, in YP_749275.1, α-helices α2 and α3 are substantially shortened (from four and five to one and 2–3 helical turns, respectively) and α3 is rotated by ∼90° from its orientation in YP_001095227.1. In addition, α3 shifts by ∼5 Å towards α2 and thus eliminates the cavity (Fig. 2 ▶). Residues from α2 (Trp71, Leu74 and Leu78) and α3 [Trp98, Val102 (Ile in YP_749275.1), Trp105 and Phe106], which are exposed to solvent in YP_001095227.1, relocate into the protein interior.

### 3.3. Dimerization mode

**Figure 3:**
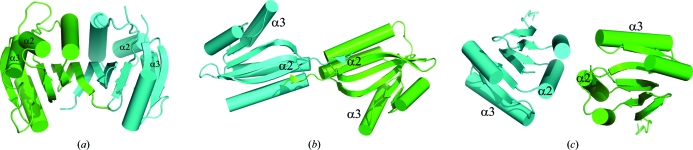
Dimerization modes. (*a*) Monomers in YP_749275.1 assemble with β-sheets lining up side by side to form a large, extended β-sheet. (*b*) YP_001095227.1 has a different dimerization mode with a more limited buried surface area. (*c*) Possible alternate mode of dimer formation in YP_001095227.1. Helices α2 and α3 are labeled.

Size-exclusion chromatography supports the assignment of a dimer as the main oligomeric state in solution for both YP_749275.1 and YP_001095227.1. In the ‘closed’ structure of YP_749275.1, the two monomers are arranged side by side and create an extended intermolecular β-sheet (Fig. 3 ▶ *a*). This interface includes additional con­tacts between the adjacent α1 helices and buries a surface area of 1439 Å^2^ with a free energy of dissociation (Δ*G* ^diss^) of 67.4 kJ mol^−1^ as calculated by the *PISA* server (Krissinel & Henrick, 2007 ▶).

The dimerization mode of the ‘open’ structure, YP_001095227.1, is different and comparatively weaker. The *PISA* server predicts two dimerization modes with similar buried surface areas. In one mode (Fig. 3 ▶ *b*), the two protein monomers associate through their N-­terminal β-strands, β2, α1 and the loop between β3 and α2, burying a surface area of only 787 Å^2^ (Δ*G* ^diss^ = 3.8 kJ mol^−1^). In the other mode (Fig. 3 ▶ *c*), dimer association would be mediated through α2 and the loop between α2 and β4, burying a surface area of 743 Å^2^ (Δ*G* ^diss^ = 0.8 kJ mol^−1^). The low values of Δ*G* ^diss^ and buried surface area suggest that these dimers may not be stable (Krissinel & Henrick, 2007 ▶) and may represent a crystal-packing artifact.

### 3.4. Distribution of conserved residues

**Figure 4:**
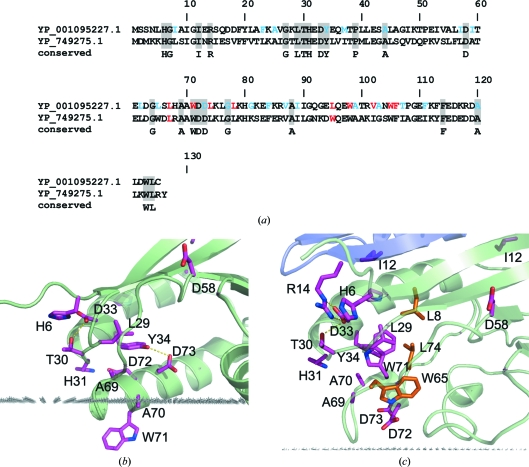
Conserved residues in YP_749275.1 and YP_001095227.1. (*a*) A sequence alignment with other members of the PFAM PB000640 family (not shown) reveals several conserved residues (marked in grey boxes). Residues from the binding cavity are colored blue and residues that are predicted to penetrate to the lipid bilayer are colored red. These residues are indicated on the structure in (*b*) for YP_001095227.1 and (*c*) for YP_749275.1. The main cluster of conserved residues from strand β1 and helices α1 and α2 is shown in purple. The protein backbone is shown in a cartoon representation. The calculated membrane boundary is shown by grey dots. A few additional nonconserved residues involved in hydrophobic interactions (Leu8, Leu74 and Trp65) in YP_749275.1 are shown in orange.

YP_749275.1 and YP_001095227.1 are assigned to family PB000640 in Pfam-B. This family includes 119 proteins from bacteria and archaea consisting of a single domain and an additional bacterial protein that includes a fusion to a universal stress protein (UspA) domain at its C-terminus. A set of conserved residues in the family was identified by aligning 20 of the most closely related bacterial proteins (>25% sequence identity in pairwise comparisons over the full length of the proteins; Fig. 4 ▶ *a*).

These conserved residues form two clusters in the protein structure. The larger cluster (Figs. 4 ▶ *b* and 4 ▶ *c*) is located in the ‘switch region’ where the two orthologs adopt distinct conformations. This cluster includes residues from β1 (His6, Gly7), α1 (Gly27, Leu29, Thr30, His31 and Tyr34) and α2 (Ala69, Ala70, Trp71, Asp72, Asp73 and Gly77). It is noteworthy that some conserved residues appear to stabilize the ‘closed’ conformation (Fig. 4 ▶ *c*), whereas others stabilize the ‘open’ conformation. For example, in the ‘closed’ state, Trp71 is buried from the solvent and participates in multiple van der Waals interactions with surrounding aromatic and aliphatic residues (Leu8, Leu29, Tyr34, Leu74 and Trp65), while His6 and His31 face the solvent and are not involved in any stabilizing hydrogen bonds. Conversely, in the ‘open’ state, Trp71 is solvent-exposed and may participate in protein–membrane interactions, whereas His6 and His31 form stabilizing hydrogen bonds with Asp33 and Asp72, respectively. In the open conformation, two other conserved residues, Tyr34 and Asp73, are located inside the hydrophobic cavity and are hydrogen bonded to each other (Fig. 4 ▶ *b*). The conservation of these residues indicates their functional importance and suggests that they may be involved in hydrogen bonding and/or ionic interactions with a bound ligand.

This ‘switch-region’ cluster is supplemented by two residues from the adjacent subunit in the dimer (Ile12 and Arg14 in the β1 strand). Arg14 of one subunit hydrogen bonds to Asp33 of the other subunit, which is likely to provide some stability and specificity to the dimer formation (YP_749275.1). Ile12 engages in hydrophobic interactions with Met40 (Val40 in YP_001095227.1). However, Arg14 does not form any contacts in the ‘open’ monomeric structure (YP_001095227.1). Thus, the conserved Ile12 and Arg14 may contribute to stabilization of the dimeric state of the protein in solution.

The second cluster consists of residues from β4 (Ala88 and Gly91), β5 (Phe114) and α4 (Ala120, Trp123 and Leu124) that aid the interaction of α4 with the β-sheet.

### 3.5. Comparison with other structures

Based on the *SCOP* classification, the SpoIIAA-like fold consists of two structural superfamilies: (i) the bacterial sporulation anti-sigma factor antagonist SpoIIAA superfamily that contains the STAS domain (PF01740 in Pfam) and (ii) the CRAL-TRIO superfamily of eukaryotic carriers of nonpolar substances (PF03765 and PF00650 in Pfam).

YP_749275.1 and YP_001095227.1 have both been assigned to a novel Sfri0576-like family in the SpoIIAA-like superfamily in *SCOP*. However, their structures can be aligned, without significant insertions or deletions, with structures from both superfamilies, although a higher *DALI* *Z* score was obtained for SpoIIAA proteins (Holm & Sander, 1995 ▶). Structural alignment using the *SSM* server (http://www.ebi.ac.uk/msd-srv/ssm/) of YP_001095227.1 (PDB code 2q3l) with SpoIIAA from *Bacillus sphaericus* (PDB code 1h4z; Seavers *et al.*, 2001 ▶) gave an r.m.s.d. of 2.6 Å over 99 C^α^ atoms. Alignment with CRAL-TRIO domains, such as α-tocopherol transfer proteins (αTTPs), led to almost the same r.m.s.d. Superposition of the corresponding ‘open’ structures (YP_001095227.1 and PDB entry 1oiz; Meier *et al.*, 2003 ▶) yielded an r.m.s.d. of 3.0 Å over 104 C^α^ atoms, while superposition of the ‘closed’ structures (YP_749275.1 and PDB entry 1r5l; Min *et al.*, 2003 ▶) resulted in an r.m.s.d. of 2.9 Å over 95 C^α^ atoms. The close superposition of these entire domains indicates a possible common evolutionary origin for all these proteins.

Despite their structural similarity, the sequence identity between either YP_749275.1 or YP_001095227.1 and proteins in the SpoIIAA-like fold families is <15%. Comparison of the sequences of different STAS domains (PDB codes 1h4z, 1til, 1tid, 1sbo, 1auz, 2vy9 and 3f43; Seavers *et al.*, 2001 ▶; Masuda *et al.*, 2004 ▶; Etezady-Esfarjani *et al.*, 2006 ▶; Kovacs *et al.*, 1998 ▶; Marles-Wright *et al.*, 2008 ▶) identified a **G** *x* **L** *x* **H** motif in some of these proteins (Seavers *et al.*, 2001 ▶). A similar motif is conserved in YP_749275.1 and YP_001095227.1 (^27^ **G**K**L**T**H**). Although the Gly and Leu residues play a structural role in providing the tight turn between the β1-strand and α2-helix, the conservation of His in the STAS-domain proteins cannot easily be explained. On the other hand, the phosphorylatable serine that is conserved in all SpoIIAA (Ser58 in 1auz, Ser57 in 1h4x; Seavers *et al.*, 2001 ▶) is substituted by negatively charged Glu/Asp residues in the majority of other members of the PB000640 Pfam-B family, including YP_749275.1 (Asp66), although Ser is present in YP_001095227.1 (Ser66). This key serine is located at the start of helix α2 and participates in the interaction of SpoIIAA with SpoIIAB and a nucleotide ligand. In the presence of ADP, the SpoIIAA–SpoIIAB complex is stable, while in the presence of ATP, SpoIIAA becomes phosphorylated and then dissociates (Aravind & Koonin, 2000 ▶; Najafi *et al.*, 1996 ▶). The lack of conservation of this serine in YP_749275.1 indicates a possible loss of functional similarity to SpoIIAA.

The YP_749275.1 and YP_001095227.1 structures suggest that these proteins can adopt open and closed conformations (Fig. 2 ▶). This situation differs from bacterial STAS proteins, the structures of which are essentially ‘closed’ with α2 and α3 tightly packed, occluding any possible cavity formation; an open state has not yet been observed in STAS-domain proteins.

**Figure 5:**
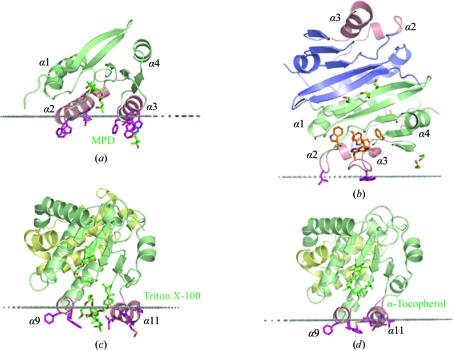
Comparison of the putative membrane association of YP_001095227.1 and YP_749275.1 with that of the CRAL-TRIO domain of human α-tocopherol transfer protein (α-­TTP). (*a*) YP_001095227.1 in the ‘open’ conformation (PDB code 2q3l). (*b*) Dimeric form of YP_749275.1 in the ‘closed’ conformation (PDB code 2ook). The second molecule is colored blue and the lid helices enclosing the binding cavity are colored pink. Residues that move from the surface to the protein interior during the conformational switch are colored orange. (*c*) Human α-TTP in the ‘open’ conformation (PDB code 1oiz; Meier *et al.*, 2003 ▶). The N-CRAL-TRIO domain is colored yellow and the lipid-binding CRAL-TRIO domain is colored green. Molecules of detergents or bound ligands are colored in dark green here and in (*d*). (*d*) α-TTP in the ‘closed’ conformation (PDB code 1r5l; Min *et al.*, 2003 ▶). In all figures, residues that penetrate or are proposed to penetrate the lipid bilayer are colored purple. Calculated boundaries between lipid head groups and the acyl-chain region are shown by grey dots.

The presence of a deep cavity in YP_001095227.1 is similar to the eukaryotic Sec14-like proteins, which also adopt the same SpoIIAA-like fold. Sec14-like proteins have a lipid-binding CRAL-TRIO domain that participates in the transport of hydrophobic substances such as lipids or α-tocopherol. Like YP_749275.1 and YP_001095227.1, yeast and human Sec14 proteins (PDB codes 1aua, 1oiz, 1r5l, 1o6u and 3b7n; Sha *et al.*, 1998 ▶; Meier *et al.*, 2003 ▶; Min *et al.*, 2003 ▶; Stocker *et al.*, 2002 ▶; Schaaf *et al.*, 2008 ▶) have been crystallized in two alternative conformations which differ in the relative disposition of two helices located at the entrance to a hydrophobic ligand-binding cavity. In particular, human αTTP (PDB code 1oiz) was obtained in a detergent-bound ‘open’ structure (Meier *et al.*, 2003 ▶) with the ‘lid’ helices α9 and α11 moved apart (Fig. 5 ▶ *c*) as well as in a ligand-bound ‘closed’ structure (PDB code 1r5l; Min *et al.*, 2003 ▶) with α11 shifted towards α9 and blocking the entrance to the cavity (Fig. 5 ▶ *d*).

### 3.6. Predicted protein–membrane association

Calculations using the *PPM* method (Lomize *et al.*, 2006 ▶) show that the ‘open’ conformation of YP_001095227.1 can associate with the lipid bilayer by immersing its exposed nonpolar residues from α2 and α3 into the lipid acyl-chain region. The predicted depth of residue penetration into the hydrophobic core of the membrane is 6.5 ± 0.4 Å and the calculated water–membrane transfer energy (Δ*G*_transf_) is −47.3 kJ mol^−1^. However, the corresponding protein membrane binding energy is expected to be smaller than the transfer energy, since part of the transfer energy must be spent on protein conformational change. The lipid-interaction residues include Leu67, Trp71, Leu74 and Leu78 from α2, and Leu95, Trp98, Val102, Trp105 and Phe106 from α3 (Fig. 5 ▶ *a*).

In contrast, the ‘closed’ conformation of YP_749275.1 was pre­dicted to form a stable dimer by the *PISA* server, as shown in Fig. 5 ▶(*b*). However, this dimer is visibly asymmetric as α3 is longer by one helical turn in subunit 2 compared with subunit 1. Furthermore, two hydrophobic residues (Leu67 in α2 and Trp95 in α3) are solvent-exposed in one subunit (indicated in purple) but buried from solvent in another subunit. These two solvent-exposed nonpolar residues may anchor the protein at the hydrophobic boundary of the lipid bilayer (Fig. 5 ▶ *b*). However, the calculated depth of penetration is only 1.2 Å and Δ*G*_transf_ is only −12.6 kJ mol^−1^, indicating a weak association.

### 3.7. Predicted protein–protein interactions

A genomic neighborhood search performed using *STRING* (http://string.embl.de) relates both YP_749275.1 and YP_001095227.1 to a TonB-dependent receptor precursor (a co-occurrence in the same species) and a universal stress protein (UspA) domain (localization in the close genetic neighborhood). One of the homologous proteins (UniProt ID Q083D4_SHEFN) from PfamB family PB000640 is fused to a UspA domain. A *PSI-BLAST* (Altschul *et al.*, 1997 ▶) search returns about three dozen bacterial proteins (mostly proteobacterial) using a sequence-identity cutoff of 25%. Most of these proteins have no functional annotation. A few contain a UspA domain, including Q083D4_SHEFN, albeit with low *e*-value scores. Although the neighborhood-matching and the *BLAST* search scores are not significant enough to confer a definitive link between these proteins, they may suggest a role of these proteins in the stress-response pathway.

## 4. Discussion

YP_749275.1 and YP_001095227.1 can now be assigned to a new bacterial protein family which adopts the SpoIIAA-like structural fold. The structures suggest that these proteins are metamorphic, adopting two distinct conformations (open and closed) which are stabilized under different environmental conditions (Murzin, 2008 ▶). We suggest that the predicted anchoring of YP_749275.1 and YP_001095227.1 to the lipid bilayer *via* helices α2 and α3 would induce a switch from the ‘closed’ to the ‘open’ conformation. Both proteins presumably exist as stable water-soluble dimers in their ‘closed’ conformation which can weakly associate with the lipid bilayer *via* nonpolar residues from the ‘lid’ helices (Fig. 5 ▶ *b*). Mem­brane binding would promote protein activation owing to the rearrangement of the ‘lid’ helices and subsequent dimer dissociation. The membrane-associated protein in the ‘open’ conformation (Fig. 5 ▶ *a*) may then bind amphiphilic ligands which have accumulated at the membrane interface.

The cellular localization of YP_749275.1 and YP_001095227.1 is currently unknown. We suggest that these proteins are cytoplasmic based on the following observations. Firstly, proteins from the same PfamB family are found in Gram-positive bacterial and archaeal species that lack the outer membrane and periplasmic space. Secondly, YP_001095227.1 has a single Cys125 that is likely to remain reduced in the bacterial cytoplasm (Ritz & Beckwith, 2001 ▶). Finally, one protein from the family is fused with the UspA domain, which is a cytoplasmic protein involved in the stress-response pathway.

We further suggest that YP_749275.1 and YP_001095227.1 may function either as water-soluble carriers of hydrophobic compounds, similar to the related CRAL-TRIO domains, or as interfacially activated enzymes. If these proteins are ligand carriers, then they can dissociate from the membrane in their ligand-loaded state. If these proteins are interfacially activated enzymes, they would remain membrane-associated while performing their chemical reactions. The presence of conserved Tyr34 and Asp73 residues at the entrance to the hydrophobic cavity may indicate possible enzymatic activity rather than simply the formation of a hydrogen bond to a ligand. For example, similar pairs of hydrogen-bonded residues (usually a Tyr–Glu pair) are found in a glycoside hydrolase (PDB code 2fhr; Watts *et al.*, 2006 ▶) and in glycosyltransferases (PDB code 1s2g; Anand *et al.*, 2004 ▶). In these enzymes, the Tyr and Glu residues form a nucleophile that interacts with the OH group of the substrate.

The natural ligands for these bacterial proteins remain unknown. The shape and the hydrophobic character of the cavity indicate a binding site for relatively large and poorly soluble compounds such as flavins, naphthoquinones or other substituted heterocycles. It is noteworthy that riboflavin and menaquinone are important for the growth of *Shewanella* cells on poorly soluble minerals, as they participate in the electron transfer to low-potential electron acceptors (Newman & Kolter, 2000 ▶; Marsili *et al.*, 2008 ▶). *Shewanella* produces significant amounts of flavins (riboflavin and riboflavin-5′-phosphate) that mediate extracellular electron transfer, leading to reduction, chelation and uptake of ferric iron by the cells (Marsili *et al.*, 2008 ▶). The uptake of iron complexes (with riboflavin or hydroxamate) can be facilitated by the TonB-dependent transport system (Schauer *et al.*, 2008 ▶). Therefore, the genomic neighborhood link between YP_749275.1/YP_001095227.1 and the TonB receptor (which also has a high co-occurrence with the nicotinamide mononucleotide transporter PnuC) may be of functional significance. In addition, the connection with UspA domains suggests a possible role of these proteins in the stress-response pathway.

Additional information about YP_749275.1 and YP_001095227.1 is available from *TOPSAN* (Krishna *et al.*, 2010 ▶) at http://www.topsan.org/explore?PDBid=2ook and http://www.topsan.org/explore?PDBid=2q3l.

## Supplementary Material

PDB reference: YP_749275.1, 2ook, r2ooksf

PDB reference: YP_001095227.1, 2q3l, r2q3lsf

## Figures and Tables

**Table 1:** Summary of crystal parameters, data collection and refinement statistics for YP_749275.1 and YP_001095227.1 Values in parentheses are for the highest resolution shell.

Protein (PDB code)	YP_749275.1 (2ook)			YP_001095227.1 (2q3l)		
Space group	*C*2			*C*222_1_		
Unit-cell parameters (Å, °)	*a* = 80.58, *b* = 40.418, *c* = 78.05, β = 92.2			*a* = 40.39, *b* = 113.96, *c* = 130.69		
Data collection	λ_1_ Se	λ_2_ Se	λ_3_ Se	λ_1_ Se	λ_2_ Se	λ_3_ Se
Wavelength (Å)	0.9184	0.9791	0.9788	0.9184	0.9793	0.9790
Resolution range (Å)	28.6–1.80 (1.85–1.80)	28.6–1.80 (1.85–1.80)	28.6–1.85 (1.90–1.85)	28.7–2.25 (2.31–2.25)	28.7–2.25 (2.31–2.25)	28.7–2.25 (2.31–2.25)
No. of observations	85996 (6294)	85818 (6269)	79298 (5823)	104546 (7445)	104441 (7352)	105302 (7527)
No. of unique reflections	22625 (1640)	22618 (1639)	20914 (1518)	14575 (1060)	14580 (1043)	14628 (1053)
Completeness (%)	96.5 (95.3)	96.5 (95.0)	96.5 (95.7)	99.0 (100.0)	98.9 (100.0)	98.9 (100.0)
Mean *I*/σ(*I*)	13.6 (2.1)	13.3 (1.7)	13.1 (1.9)	17.8 (3.6)	17.2 (3.1)	16.4 (2.8)
*R*_merge_ on *I*†	0.056 (0.653)	0.056 (0.763)	0.059 (0.672)	0.067 (0.467)	0.071 (0.546)	0.075 (0.629)
*R*_meas_ on *I*‡	0.065 (0.758)	0.066 (0.886)	0.069 (0.781)	0.072 (0.504)	0.077 (0.588)	0.081 (0.678)
Model and refinement statistics						
Data set used in refinement	λ_1_ MAD Se			λ_1_ MAD Se		
Cutoff criterion	|*F*| > 0			|*F*| > 0		
*R*_cryst_§	0.183			0.181		
*R*_free_¶	0.233			0.239		
Resolution range (Å)	28.6–1.80			28.7–2.25		
No. of reflections (total)	22625			14570		
No. of reflections (test set)	1162			754		
Completeness (%)	96.5			98.8		
Stereochemical parameters						
Restraints (r.m.s.d. observed)						
Bond lengths (Å)	0.012			0.017		
Bond angles (°)	1.53			1.76		
Average isotropic *B* value (Å^2^)	35.4			51.3		
ESU†† based on *R*_free_ value (Å)	0.141			0.216		
Protein residues/atoms	247/2026			248/1962		
Water molecules/other solvent molecules	224/6			95/13		

† *R*_merge_ = 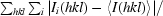 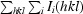.

‡ *R*_meas_ = 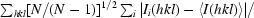 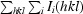 (Diederichs & Karplus, 1997 ▶).

§ *R*_cryst_ = 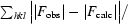 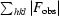, where *F*_calc_ and *F*_obs_ are the calculated and observed structure-factor amplitudes, respectively.

¶ *R*_free_ is the same as *R*_cryst_, but for 5.1% (2ook) and 5.2% (2q3l) of the total number of reflections that were chosen at random and omitted from refinement.

†† Estimated overall coordinate error (Cruickshank, 1999 ▶; Collaborative Computational Project, Number 4, 1994 ▶).
